# Mass Spectrometry-Based Screening Reveals Inhibitors of Cholesterol 25-Hydroxylase

**DOI:** 10.1021/acschembio.6c00385

**Published:** 2026-06-12

**Authors:** Atikur Rahman, Elijah H. Hayes, Drew J. Adams

**Affiliations:** † Department of Genetics and Genome Sciences, 2546 Case Western Reserve University School of Medicine, Cleveland, Ohio 44106, United States; ‡ Chemical Biology Program, Case Western Reserve University School of Medicine, Cleveland, Ohio 44106, United States; § Department of Pharmacology, Case Western Reserve University School of Medicine, Cleveland, Ohio 44106, United States

## Abstract

Cholesterol 25-hydroxylase (CH25H) metabolizes cholesterol to 25-hydroxycholestrol (25HC) and plays a pathological role in osteoarthritis, Alzheimer’s Disease, and other diseases. As an ER-resident transmembrane diiron lipid oxidase, CH25H remains a challenging enzyme to study, and no small molecule inhibitors of CH25H are available. As a first step toward developing CH25H inhibitors, we established a mass spectrometry-based cellular assay monitoring CH25H-mediated production of 25HC. Screening of this assay across a focused library of over 100 small molecules containing either an iron-coordinating moiety or a sterane ring system revealed three potent inhibitors of cellular CH25H activity (U73343, Ciclopirox, and phenanthroline). We additionally developed a secondary assay of CH25H function monitoring a transcriptional response confirmed to result from 25HC production. Finally, U73343 but not the iron-binding hits showed strong selectivity versus related diiron lipid oxidases. Overall, our work establishes a series of cell-based assays monitoring CH25H function and nominates first-in-class cell-active inhibitors of this disease-relevant enzyme.

## Graphical abstract

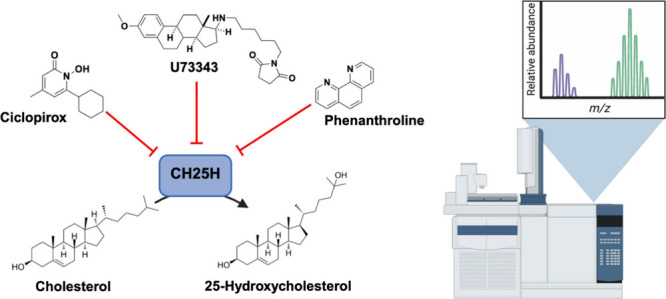

Cholesterol 25-hydroxylase (CH25H) is a member of the diiron lipid oxidases, a family of 12 human enzymes that use a nonheme diiron catalytic center to hydroxylate or desaturate sterols, fatty acids, and sphingolipids.^1,2^ Each diiron lipid oxidase is an ER-resident transmembrane protein that relies on cytochrome b5-like proteins for turnover, making them challenging to study in purified form. As a result, many diiron lipid oxidases, CH25H included, lack chemical probes.

CH25H converts cholesterol to 25-hydroxy-cholesterol (25HC), an oxysterol with pleiotropic effects that include transcriptional signaling via LXR and membrane fluidizing properties.^3,4^ CH25H is an interferon stimulated gene, and expression of CH25H is typically low outside of inflammatory contexts. CH25H KO mice are grossly normal but show compromised ability to fight viral infection, supporting 25HC as an antiviral oxysterol.^5,6^ However, additional studies have demonstrated that CH25H activity also has pro-inflammatory properties via promotion of TLR4 signaling and enhanced NFkB activation.^7,8^ These pro-inflammatory functions contribute to the pathology of numerous diseases, since CH25H KO mice are strongly protected in models of tauopathy,^9^ neuroinflammation,^10–12^ intestinal fibrosis,^13^ vascular damage,^8^ and osteoarthritis.^14^ CH25H thus plays important roles in immunity but may also be a promising therapeutic target across a wide range of human diseases.

Small-molecule probes are needed to evaluate the therapeutic efficacy and potential on-target liabilities of CH25H inhibition in disease models. As a first step toward development of CH25H inhibitors, we have established a gas chromatography/mass spectrometry (GCMS)-based assay to directly and quantitatively measure production of CH25H’s product 25HC. While this assay has moderate throughput, we applied it to two focused libraries of molecules that we imagined could inhibit CH25H function either by coordinating its catalytic iron centers or mimicking its cholesterol substrate. From a screen of over 100 molecules, we have validated 3 as cellular inhibitors of CH25H function, each with EC50 ca. 1–2 uM. We have additionally established secondary assays monitoring a transcriptional response validated to be dependent on CH25H catalytic activity^15^ and monitoring selectivity for CH25H versus two closely related sterol-metabolizing diiron lipid oxidases. Together, these studies provide the first cell-active inhibitors of CH25H and establish a screening method applicable to the discovery and validation of future CH25H inhibitors.

## Results and Discussion

As an initial path to identifying and characterizing CH25H inhibitors, we first established a gas chromatography/mass spectrometry (GCMS)-based assay of cellular CH25H activity in which HEK293T cells were transfected with a mouse Ch25h-FLAG construct. After confirming expression of Ch25h in these cells, we measured production of its product 25-hydroxycholesterol (25HC) via GCMS (see Figure 1a,b, as well as Figure S1a–d). While 25HC was undetectable at baseline in mock transfected cells, transient overexpression of Ch25h-FLAG led to clear production of 25HC (Figure 1c,d). In contrast, transfection of a mutant allele of Ch25h that disrupts two key histidine residues necessary for proper iron coordination in the catalytic center (H242Q/H243Q) did not elevate 25HC levels, further confirming Ch25h-mediated production of 25HC in this assay (Figure 1c,d). 25HC levels appeared highest at 24 h, supporting use of this time point in subsequent studies aimed at identification of Ch25h inhibitors (Figures 1e, and Figures 2 and 3 below). Comparable activity was also observed following transfection of a human CH25H-FLAG allele (but not the corresponding H242Q/H243Q mutant), highlighting the applicability of this assay system across species (see Figure 1f, as well as Figure S1e).

**Figure 1:**
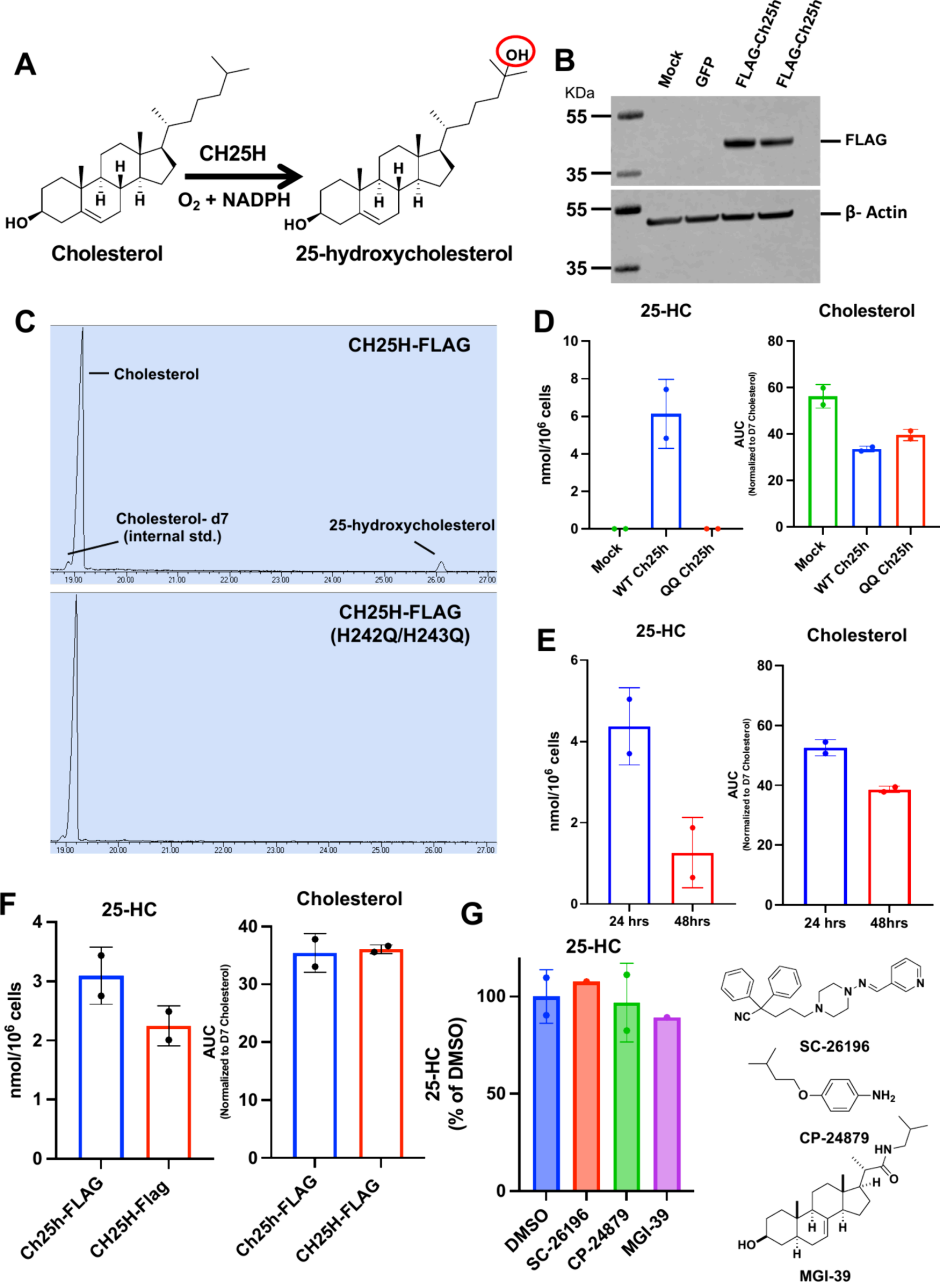
Validation and optimization of the Ch25h overexpression GCMS assay. (a) Schematic of CH25H-mediated conversion of cholesterol to 25-hydroxycholesterol (25-HC). (b) Western blot confirming expression of FLAG-Ch25h in HEK293T cells 24 h post-transfection. Two independent wells of FLAG-Ch25h-expressing cells were analyzed. (c) Representative GC-MS chromatograms showing 25-HC detection in cells expressing wild-type Ch25h-FLAG but not catalytically inactive Ch25h (H242Q/H243Q). d7-cholesterol was used as internal standard. (d) GCMS-based quantification of 25-HC and total cholesterol in cells expressing the indicated alleles of Ch25h. “Mock” refers to cells exposed to Lipofectamine but not plasmid DNA. (e) Time-course analysis of 25-HC production at 24 and 48 h post-transfection. (f) GCMS analysis of 25HC and cholesterol levels in HEK293T cells transfected with Ch25h-FLAG (mouse) or CH25H-FLAG (human). (g) GCMS-based quantitation of 25HC levels in cells transfected with Ch25h-FLAG and treated with the indicated small molecules (10 μM, 24 h). SC-26196, FADS2 inhibitor; CP-24,879, FADS1/FADS2 inhibitor; MGI39, SC5D inhibitor. In panels (d)–(f), *n* = 2 independent wells were analyzed by GCMS. In panel (g), SC-26196 and MGI-39 are *n* = 1 well, while DMSO and CP-24879 conditions are *n* = 2 independent wells.

While the diiron lipid oxidase family comprises 12 enzymes, only a minority have been targeted by small molecule inhibitors. To assess whether existing inhibitors of other family members might also inhibit Ch25h, we evaluated the ability of three structurally unrelated inhibitors (SC-26196,^16^ targeting FADS2; CP-24,879,^17^ targeting FADS1/FADS2; MGI-39,^18^ targeting SC5D) to reduce 25HC production following transfection of mouse Ch25h. Treatment with 10 μM of these molecules had no impact on 25HC production, arguing that existing diiron lipid oxidase inhibitors were unlikely to be strong starting points for inhibiting CH25H (Figure 1g).

We next sought to use small-molecule screening as an approach to identify CH25H inhibitors. While our GCMS-based assay has relatively modest throughput, it provides a direct and quantitative readout for assessing cellular CH25H inhibition. Given the iron-dependence of CH25H, we initially wondered whether a focused library of molecules known to bind iron or containing common metal coordination motifs could contain Ch25h inhibitors. We assembled an initial set of 38 known bioactive small molecules that contained such chemical motifs and evaluated their impact on 25HC production in Ch25h-expressing HEK293 cells. Using a hit threshold of 80%, we identified two hits (Figure 2a; see Table S1 for performance of all library members). Retest in dose confirmed the inhibitory effects of both ciclopirox and phenanthroline, with ciclopirox having EC_50_ ca. 2 μM and phenanthroline slightly less potent (Figure 2b,c). Ciclopirox is an FDA-approved antifungal agent that uses an *N*-hydroxypyridone moiety to coordinate iron and inhibit its target in yeast;^19^ phenanthroline is a commonly used ligand for divalent metals with limited prior application in biology. Neither molecule showed cytotoxicity, as evidenced by unchanged cholesterol levels in our GCMS assay (Figure 2b,c) and unchanged ATP levels in a separate CellTiter-Glo assay (Figure S 1f). Additionally, these molecules had little effect on the protein levels of Ch25h, and only at concentrations well above the EC_50_ for suppression of 25HC production, discounting the possibility that these molecules inhibited 25HC production by altering Ch25h expression (Figure 2d,e). Finally, we confirmed that these molecules also fully inhibited human CH25H (Figure 2f), highlighting their potential application both in human cell systems and in mouse models of disease. This screen reveals that only a low fraction of small molecules that contain known iron-binding motifs carry the ability to inhibit CH25H. We can find no reports evaluating the iron-binding affinity of Ciclopirox and phenanthroline under comparable conditions, making it challenging to assess how their intrinsic affinity for iron may contribute to their interaction with Ch25h. Notably, both Ciclopirox and phenanthroline are hydrophobic other than their iron-coordinating moieties, which may favor interaction with the presumably hydrophobic cholesterol-binding transmembrane active site of CH25H. Finally, while Ciclopirox and phenanthroline have strong potency for initial screening hits, their potency may be higher in assay settings in which CH25H is expressed at physiological levels rather than overexpressed.

**Figure 2:**
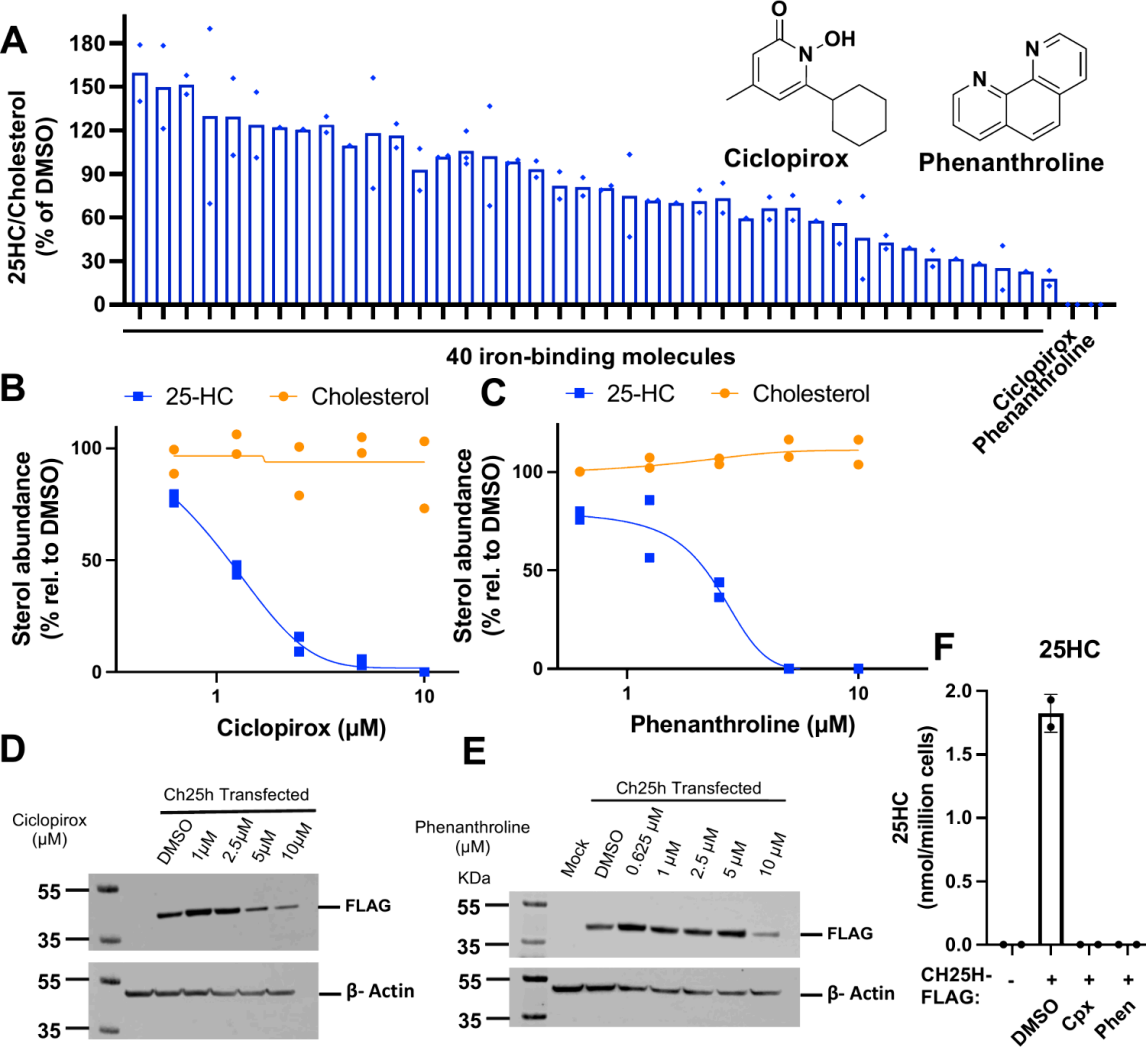
Screening reveals Ciclopirox and Phenanthroline as Ch25h inhibitors. (a) HEK293T cells transfected with Ch25h-FLAG were treated with 42 bioactive small molecules containing likely iron-coordinating moieties (10 μM) and 25HC levels were measured by GCMS after 24h. The screen was performed in duplicate, and two leading hits are drawn. (b, c) GCMS-based quantitation of 25HC and cholesterol levels in the assay from panel (a) following treatment (24 h) with the indicated concentrations of Ciclopirox (left) or phenanthroline (right). Two independent wells of each condition are presented. (d, e) Western blotting for FLAG to assess Ch25h expression levels following 24 h treatment with the indicated concentrations of Ciclopirox (d) and phenanthroline (e). (f) GCMS-based quantitation of 25HC with or without transfection with human CH25H-FLAG and treatment with Ciclopirox or phenanthroline (10 μM, 24 h). Two independent wells of each condition are presented. All experiments used HEK293T cells.

Since this low-throughput GCMS approach successfully identified cellular Ch25h inhibitors, we next wondered whether an expanded screen could identify additional series of inhibitors. Because metal coordination can promote off-target interactions with other iron-binding enzymes, we next sought to identify complementary inhibitors that might mimic Ch25h’s sterol substrate. Adopting a strategy we employed previously,^20^ we selected 78 sterol- and steroid-containing molecules from within our CWRU libraries for evaluation in our GCMS assay of 25HC production. After evaluating each molecule at 10 μM, we obtained only a single hit, U73343, which showed more than 90% inhibition of 25HC production (Figure 3a; see Table S1 for performance of all library members). U73343 is an inactive analogue of the phospholipase C inhibitor U73142 and was previously shown to inhibit the sterol 14-reductase enzyme in cholesterol biosynthesis.^20,21^ Subsequent confirmation in dose revealed an EC_50_ of ca. 1 μM for U73343, comparable to ciclopirox (Figure 3b). No cytotoxicity was observed for U73343 until concentrations above 10 μM, and no effect on CH25H protein levels was observed up to 10 μM (see Figure 3c, as well as Figure S1f). Additionally, U73343 also fully inhibited human CH25H (Figure 3d)

**Figure 3:**
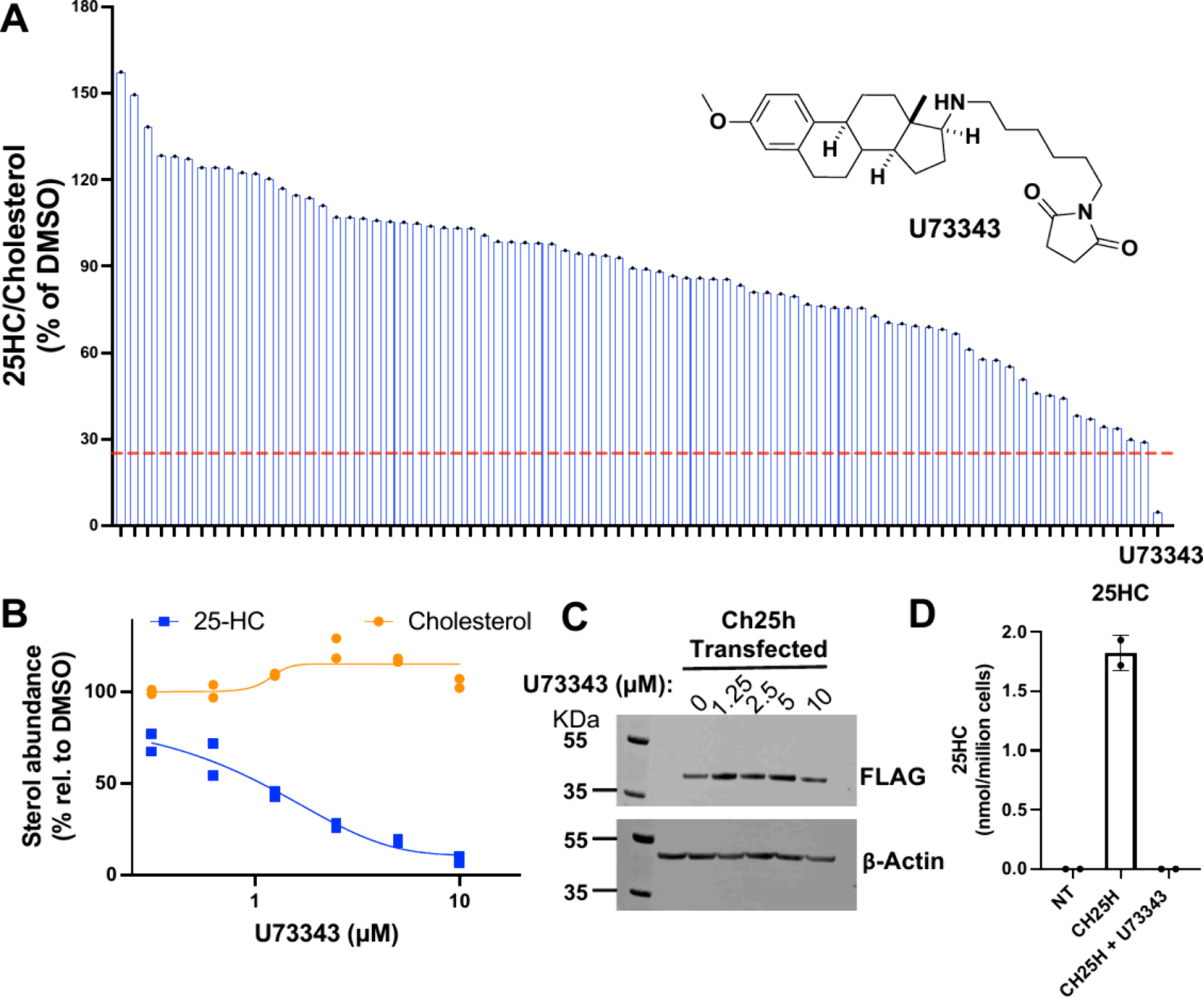
Screening of a steroid-focused library reveals U73343 as a Ch25h inhibitor. (a) HEK293T cells transfected with Ch25h-FLAG were treated with 78 bioactive small molecules containing sterane or steroid moieties (10 μM) and 25HC levels were measured in singlicate by GCMS after 24 h. (b) Quantitation of 25HC and cholesterol levels in Ch25h-FLAG-transfected cells following treatment (24 h) with the indicated concentrations of U73343. Two independent wells of each condition are presented. (c) Western blotting for FLAG to assess Ch25h expression levels following 24 h treatment with the indicated concentrations of U73343. (d) Quantitation of 25HC with or without transfection with human CH25H-FLAG and treatment with U73343 (24 h, 10 μM). Two independent wells of each condition are presented. All experiments used HEK293T cells.

Next, we evaluated the selectivity of our three validated CH25H inhibitors versus other diiron lipid oxidase enzymes. As an initial test, we evaluated SC4MOL (MSMO1) and SC5D, two diiron lipid oxidases essential for de novo synthesis of cholesterol. These enzymes were chosen due to their close phylogenetic relation to CH25H and their ease of analysis by GCMS,^18,22^ providing a parallel detection method to our CH25H screening assay.^23^ Using GCMS-based quantitation of SC4MOL and SC5D substrates, ciclopirox and phenanthroline showed partial inhibition of SC4MOL activity and substantial inhibition of SC5D (Figure 4a,b). In contrast, U73343 showed no inhibition of these enzymes at concentrations up to 10 μM (Figure 4a,b). This high selectivity versus closely related diiron lipid oxidases suggests U73343 may be a relatively potent and selective starting point for optimization of improved CH25H inhibitors. U73343’s aminosteroid structure also offers reasonable physicochemical properties (466 Da, log *D* = 3.3, CNS MPO score = 3.5; properties calculated by Collaborative Drug Discovery software), supporting its potential as a starting point for further optimization.

**Figure 4:**
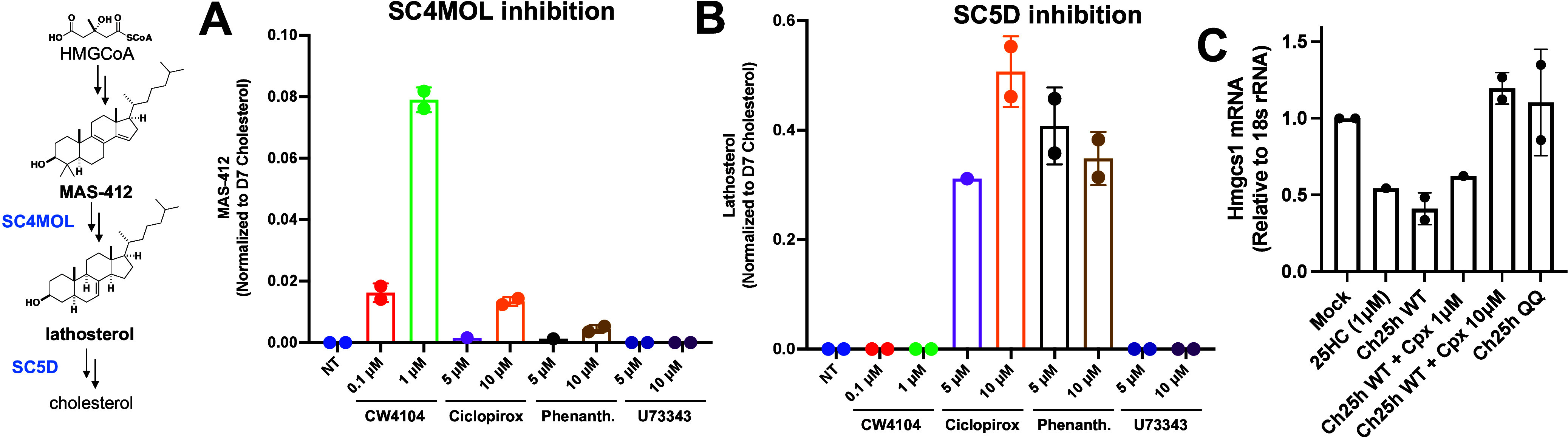
Characterization of CH25H selectivity and CH25H-dependent transcriptional effects. (a) GCMS-based quantitation of the SC4MOL substrate MAS-412 in DLD1 cells treated with the indicated concentrations of four small molecules. CW4104 is an established SC4MOL inhibitor. NT, nontreated. (b) As in panel (a), but the SC5D substrate lathosterol is quantitated. Both SC4MOL and SC5D are diiron lipid oxidases that, like CH25H, metabolize sterols. c) RT-qPCR analysis of *Hmgcs1* transcript levels in CHO cells treated as indicated. Cpx: Ciclopirox. Ch25h QQ: inactive mutant Ch25h allele. Two independent wells were analyzed in each condition except in panel (c), in which the 25HC and Cpx 1 μM conditions are *n* = 1.

Finally, we sought to establish that CH25H inhibition could abrogate cellular responses known to depend on CH25H-mediated 25HC production. As an initial approach, we assayed a previously reported transcriptional response in which CH25H upregulation represses *Hmgcs1* expression in CHO cells.^15^ We first confirmed that transient transfection of Ch25h-FLAG in CHO cells was sufficient to repress *Hmgcs1* expression (Figure 4c). Importantly, the inactive QQ allele of Ch25h did not lower *Hmgcs1* transcript levels, highlighting that this phenotype is dependent on Ch25h catalytic activity (Figure 4c). As previously reported, supplementation with purified 25HC also repressed *Hmgcs1* expression, further supporting 25HC production as central to this transcriptional response (Figure 4c).^15^ We then evaluated Ciclopirox as a representative potent CH25H inhibitor. Gratifyingly, Ciclopirox dose-responsively enhanced *Hmgcs1* expression in Ch25h-expressing CHO cells, highlighting its ability to suppress an established Ch25h-dependent transcriptional response (Figure 4c). Together, these data establish the first cell-active CH25H inhibitors and provide a new screening approach for the identification and validation of future CH25H inhibitors.

## Materials and Methods

### Reagents and Constructs

#### Small Molecules and Lipids

All small molecule inhibitors and lipid standards were procured from commercial vendors. SC-26196, CP-24879, Ciclopirox, and U73343 were purchased from MedChem Express; MGI39 was a gift of F. Bracher (Ludwig Maximilians Universitat Munchen). The quantitative lipid standard 25-hydroxycholesterol (25HC) and the screening compound 1,10-Phenanthroline were obtained from Cayman Chemical. Cholesterol-d7, utilized as the internal standard for gas chromatography–mass spectrometry (GC-MS) analyses, was sourced from Avanti Polar Lipids, Inc.. The focused screening libraries, used to identify novel CH25H inhibitors via the GC-MS assay, comprised two distinct sets of compounds: a library of 42 known iron-chelating agents and a library of 78 sterol analogues and lipid-targeting inhibitors. These compounds were derived from a merger of the Sigma LOPAC_1280_ and Selleck Bioactive Compound Library L1700 collections. All compounds were prepared in dimethyl sulfoxide (DMSO) stock solutions and stored at −20 °C.

#### Antibodies and Expression Vectors

The C-terminal FLAG-tagged mouse cholesterol 25-hydroxylase (Ch25h) wild-type expression vector (NM_009890) was purchased from Origene (Catalog No. MR225185). The catalytically inactive mouse Ch25h construct (inactive Ch25h; H242Q/H243Q) was generated from this wild-type plasmid using the QuikChange Lightning Site-Directed Mutagenesis Kit (Agilent Technologies) according to the manufacturer’s protocol. Mutagenic primers used were: forward 5′-TTGCAGTTAAA­CTGAGATTGTT­GCATGTCGTGG­TGAGCCA-3′ and reverse 5′-TGGCTCACCA­CGACATGCAA­CAATCTCAGTTT­AACTGCAA-3′. Successful introduction of the H242Q/H243Q mutations was confirmed by Sanger sequencing. For human CH25H, a C-terminal FLAG-tagged inactive human CH25H construct (H242Q/H243Q) was obtained from VectorBuilder. Wild-type human CH25H-FLAG was then generated from this inactive construct by site-directed mutagenesis using the QuikChange Lightning Site-Directed Mutagenesis Kit (Agilent Technologies). Mutagenic primers used were: forward 5′-TGCAGTTAAA­GTGAGAGTGA­TGCAGGTCGT­GGTGCACC-3′ and reverse 5′-GGTGCACCACG­ACCTGCATCA­CTCTCACTTT­AACTGCA-3′. Successful reversion to wild-type sequence was confirmed by Sanger sequencing. For Western blot applications, the ANTI-FLAG M2 mouse monoclonal antibody was purchased from Millipore Sigma (Catalog No. F1804).

#### Cell Culture

HEK293T cells were maintained in Dulbecco’s Modified Eagle Medium (DMEM) supplemented with 10% fetal bovine serum (FBS) and 1% penicillin/streptomycin (Corning, MT30002CI). DLD1 cells (CCL-221) purchased from ATCC, were cultured in RPMI1640 medium supplemented with 10% FBS, 1% penicillin/streptomycin (Corning, MT30002CI) and 10 mM HEPES buffer (Gibco, 15630080). CHO cells were maintained in Ham’s F-12 nutrient mixture supplemented with 10% fetal bovine serum (FBS) and 1% penicillin/streptomycin. All cell lines were incubated at 37 °C in a humidified atmosphere containing 5% CO_2_. Cells were routinely tested for mycoplasma contamination.

#### Plasmid Transfection

To establish a functional cholesterol 25-hydroxylase (CH25H) overexpression system, HEK293T cells were transiently transfected with the CH25H-FLAG or Ch25h-FLAG expression plasmids. For transfection, HEK293T cells were seeded in 6-well plates at a density of 1 × 10^6^ cells per well and were allowed to adhere overnight. The following day, cells were transfected using Lipofectamine 2000 reagent (Thermo Fisher Scientific, Catalog No. 11668027) according to the manufacturer’s protocol. The standard protocol was employed, and the medium was replaced with fresh, supplemented growth medium after a 5-h incubation. Following the medium change, cells were incubated for an additional 19 h post-transfection to achieve peak protein expression and maximal CH25H enzymatic activity prior to conducting downstream functional assays.

### GC-MS Analysis and Inhibitor Screening

#### Functional Screening of Ch25h Activity in HEK293T Cells

HEK293T cells were seeded at a density of 1 × 10^6^ cells per well in 6-well plates and incubated overnight. The following day, cells were transiently transfected with the Ch25h-FLAG expression plasmid. Five hours post-transfection, the media was replaced with fresh, supplemented growth media. Cells were then immediately treated with the respective compounds from the focused libraries described above at a single screening concentration of 10 μM or with vehicle control (DMSO). The total incubation period post-transfection was 24 h.

#### Lipid Extraction and Saponification

Following the 24-h incubation, cells were placed on ice, media was aspirated, and the cells were washed three times with ice-cold phosphate-buffered saline (PBS). The plates were immediately stored at −80 °C for at least 10 min (or overnight) to ensure complete cell lysis. The frozen plates were then thawed at RT for 10 min. Lipid extraction was initiated by adding 1 mL of 3:2 (v/v) hexane:2-propanol mixture to each well. Cholesterol-d7 (5 μg/mL in hexane), which serves as the internal standard to control for extraction and injection variability, was added to the extract at a final volume of 100 μL. After a 10 min incubation at RT with gentle agitation, the samples were transferred to glass vials. This extraction process was repeated three times to maximize sterol recovery. The combined extracts were dried down under a nitrogen stream.

The lipid residue was then subjected to alkaline saponification to quantify total sterol content. This was performed by adding 1 mL of 100% ethanol followed by 300 μL of 10 N KOH (prepared in 75% EtOH, v/v). The samples were sealed and heated at 80 °C for 1 h. The resulting solution was then neutralized, dried under N_2_ and resuspended in 500 μL of chloroform and 500 μL of double-distilled water (ddH_2_O). The organic phase containing the sterols was transferred to a clean GC vial and evaporated to dryness under N_2_.

#### Sterol Derivatization and Instrumental Analysis

The dried sterol residue was derivatized by adding 100 μL of *N*-methyl-*N*-(trimethylsilyl) trifluoroacetamide (MSTFA) (Sigma–Aldrich Chemical Co., Catalog Number 69479). The capped GC vials were heated at 80 °C for 1 h to generate di-TMS-25-hydroxycholesterol. Samples were then transferred to a GC glass insert for analysis.

Two μL of each derivatized sample were analyzed by GC-MS using an Agilent 5973 Network Mass Selective Detector equipped with a 6890-gas chromatograph system and a HP-5MS capillary column (60 m × 0.25 mm × 0.25 μm). Samples were injected in splitless mode, and analysis was performed using electron impact ionization. Peaks corresponding to diagnostic ion fragments were integrated for quantification: cholesterol-d7 (*m*/*z* 375), cholesterol (*m*/*z* 368), 25-hydroxycholesterol (*m*/*z* 546), dihydro-T-MAS (*m*/*z* 486), lathosterol (*m*/*z* 458). Calibration curves were generated by injecting varying concentrations of the respective sterol standards along with a fixed amount of cholesterol-d7 to ensure linearity and accuracy across the quantitative range.

#### Cell Viability Assay

HEK293T cells were first seeded in 6-well plates (1 × 10^6^ cells per well) and transiently transfected with the Ch25h-FLAG expression plasmid. Five hours post-transfection, the media was replaced, and cells were harvested, counted, and reseeded into white, clear-bottom 384-well plates at a density of 1,000 cells per well. The Ch25h-overexpressing cells were immediately treated with a 8-point, 1:2 serial dilution of the test compounds: Ciclopirox, 1,10-Phenanthroline monohydrate, and U73343, starting at a maximum concentration of 100 μM. Vehicle-only control wells were treated with DMSO (≤0.1% final concentration). Cells were incubated for 24 h at 37 °C. Following incubation, cellular adenosine triphosphate (ATP) levels were quantified using the CellTiter-Glo (CTG) luminescent assay (Promega, G7572). Five microliters (5 μL) of CTG reagent was dispensed into each well using the EL406 Microplate Washer Dispenser (BioTek). The mixture was then incubated at RT for 10 min with gentle agitation. Luminescence was calculated using the Synergy Neo2Multimode microplate reader (BioTek). Dose curves were normalized to DMSO control wells and fit using a four-parameter variable slope nonlinear regression function in GraphPad Prism.

#### Western Blot Analysis

HEK293T cells, seeded at 1 × 10^6^ cells/well, were transiently transfected with Ch25h-FLAG and subsequently treated with respective Ch25h inhibitors. Cells were pelleted, washed with ice-cold PBS, then lysed with sonication (15 × 1-s pulses) in PBS containing 1X Halt Protease and Phosphatase Inhibitor cocktail (Thermo Fisher Scientific, 78440). Protein concentrations were quantified using the Pierce BCA Assay (Thermo Fisher Scientific, 23225) with colorimetric development measured on a Biotek Synergy Neo2 microplate reader. Normalized protein samples were resolved by SDS-PAGE on 4%–12% gradient gels (Invitrogen, NW04122BOX), followed by transfer onto PVDF membranes using the iBlot2 Dry Transfer System (Thermo Fisher Scientific). Membranes were blocked with Pierce Protein-Free T20 Blocking Buffer (Thermo Fisher Scientific, 37571) and probed overnight with primary antibodies against FLAG M2 (Millipore, F1804) and beta-Actin (Sigma–Aldrich, A3854). Following incubation with HRP-conjugated secondary antibody (Cell Signaling Technologies, 7074), signal detection was performed using either SuperSignal West Pico PLUS (Thermo Fisher Scientific, 34580) or SuperSignal West Femto (Thermo Fisher Scientific, 34095) Chemiluminescent Substrate, and images were acquired on the LI-COR Odyssey Fc Imaging System.

#### RT-qPCR Analysis of Hmgcs1 Expression

CH25H-inducible CHO cells (ref 15) were seeded at 500 000 cells per well in 6-well plates and allowed to adhere overnight. The following day, cells were transiently transfected with either wild-type Ch25h-FLAG or the catalytically inactive Ch25h H242Q/H243Q mutant construct using Lipofectamine 2000, as described above. Five hours post-transfection, media was replaced and cells were treated with Ciclopirox (1 μM or 10 μM) or 25-hydroxycholesterol (25HC, 1 μM) for an additional 19 h, for a total of 24 h post-transfection. Mock-transfected cells treated with vehicle (DMSO) served as the baseline control. Total RNA was extracted using the RNeasy Mini Kit (Qiagen, Catalog No. 74106) according to the manufacturer’s protocol. RNA concentration and purity were assessed by NanoDrop spectrophotometry. cDNA was synthesized using the High-Capacity RNA-to-cDNA Kit (Applied Biosystems Catalog No. 4387406) per the manufacturer’s instructions. Quantitative PCR was performed using SYBR Green chemistry on a QuantStudio 7. Hmgcs1 expression was normalized to hamster 18S rRNA as the reference gene. The following primers were used: hamster 18S rRNA forward 5′-TAAGTCCC­TGCCCTTT­GTACACA-3′ and reverse 5′-GATCCGA­GGGCCTCA­CTAAAC-3′; hamster Hmgcs1 forward 5′-CCTATG­ACTGCATT­GGGCG-3′ and reverse 5′-CCCAGAC­TCCTCAAA­CAGCTG-3′. Relative expression was calculated using the 2^–ΔΔCt^ method, with mock-transfected DMSO-treated cells set as the reference condition.

## Supplementary Material



## Notes

Figure S1, GC-MS method development for the quantification of 25- hydroxycholesterol in cells; Table S1, Raw screening data for the iron chelator and sterol library members (PDF)

## References

[1] Cid N. G. (2017). Phylogenomic
analysis of integral diiron membrane histidine motif-containing
enzymes in ciliates provides insights into their function and evolutionary
relationships. Mol. Phylogenet. Evol..

[2] Shanklin J., Guy J. E., Mishra G., Lindqvist Y. (2009). Desaturases:
emerging models for understanding functional diversification of diiron-containing
enzymes. J. Biol. Chem..

[3] Nguyen C., Saint-Pol J., Dib S., Pot C., Gosselet F. (2024). 25-Hydroxycholesterol
in health and diseases. J. Lipid Res..

[4] Lund E. G., Kerr T. A., Sakai J., Li W. P., Russell D. W. (1998). cDNA cloning
of mouse and human cholesterol 25-hydroxylases, polytopic membrane
proteins that synthesize a potent oxysterol regulator of lipid metabolism. J. Biol. Chem..

[5] Bauman D. R. (2009). 25-Hydroxycholesterol secreted by macrophages
in response to Toll-like
receptor activation suppresses immunoglobulin A production. Proc. Natl. Acad. Sci. U. S. A..

[6] Liu S. Y. (2013). Interferon-inducible
cholesterol-25-hydroxylase broadly inhibits
viral entry by production of 25-hydroxycholesterol. Immunity.

[7] Gold E. S. (2014). 25-Hydroxycholesterol acts as an amplifier of inflammatory
signaling. Proc. Natl. Acad. Sci. U. S. A..

[8] Canfran-Duque A. (2023). Macrophage-Derived 25-Hydroxycholesterol
Promotes Vascular Inflammation,
Atherogenesis, and Lesion Remodeling. Circulation.

[9] Toral-Rios, D. Cholesterol 25-hydroxylase mediates neuroinflammation and neurodegeneration in a mouse model of tauopathy. J. Exp. Med. 2024, 221, 10.1084/jem.20232000. PMC10908359 38442267

[10] Wong M. Y. (2020). 25-Hydroxycholesterol amplifies microglial IL-1beta production in
an apoE isoform-dependent manner. J. Neuroinflammation.

[11] Romero J., Toral-Rios D., Yu J., Paul S. M., Cashikar A. G. (2024). 25-hydroxycholesterol
promotes brain cytokine production and leukocyte infiltration in a
mouse model of lipopolysaccharide-induced neuroinflammation. J. Neuroinflammation.

[12] Izumi Y. (2021). A Proinflammatory Stimulus Disrupts Hippocampal Plasticity and Learning
via Microglial Activation and 25-Hydroxycholesterol. J. Neurosci..

[13] Raselli T. (2019). The Oxysterol Synthesising
Enzyme CH25H Contributes to the Development
of Intestinal Fibrosis. J. Crohns Colitis.

[14] Choi S. H. (2024). Oral transforming growth
factor-beta receptor 1 inhibitor vactosertib
promotes osteosarcoma regression by targeting tumor proliferation
and enhancing anti-tumor immunity. Cancer Commun.
(Lond).

[15] Saito H. (2023). Hydroxylation site-specific
and production-dependent effects of endogenous
oxysterols on cholesterol homeostasis: Implications for SREBP-2 and
LXR. J. Biol. Chem..

[16] Obukowicz M. G. (1998). Novel, selective delta6 or delta5 fatty acid
desaturase inhibitors
as antiinflammatory agents in mice. J. Pharmacol.
Exp. Ther..

[17] Obukowicz M. G. (1998). Identification and characterization of a novel delta6/delta5 fatty
acid desaturase inhibitor as a potential anti-inflammatory agent. Biochem. Pharmacol..

[18] Giera M., Renard D., Plossl F., Bracher F. (2008). Lathosterol
side chain
amides: a new class of human lathosterol oxidase inhibitors. Steroids.

[19] Hu L. (2020). Development of Novel
N-hydroxypyridone Derivatives as Potential Anti-Ischemic
Stroke Agents. J. Med. Chem..

[20] Sax J. L., Hubler Z., Allimuthu D., Adams D. J. (2021). Screening Reveals
Sterol Derivatives with Pro-Differentiation, Pro-Survival, or Potent
Cytotoxic Effects on Oligodendrocyte Progenitor Cells. ACS Chem. Biol..

[21] Takenouchi T., Ogihara K., Sato M., Kitani H. (2005). Inhibitory
effects
of U73122 and U73343 on Ca^2+^ influx and pore formation
induced by the activation of P2X7 nucleotide receptors in mouse microglial
cell line. Biochim. Biophys. Acta.

[22] Pleshinger M. J. (2022). Inhibition of SC4MOL
and HSD17B7 shifts cellular sterol composition
and promotes oligodendrocyte formation. RSC
Chem. Biol..

[23] Hubler Z. (2018). Accumulation of 8,9-unsaturated
sterols drives oligodendrocyte formation
and remyelination. Nature.

