# Supplementary material for: Mass Spectrometry-Based Screening Reveals Inhibitors of Cholesterol 25-Hydroxylase
Source: ACS Chem Biol. 2026 Jun 12;21(7):1623–30. doi: 10.1021/acschembio.6c00385 (PMC13386465; doi:10.1021/acschembio.6c00385)
Supplement: Supplementary file 1 [file cb6c00385_si_001.pdf]

SUPPORTING INFORMATION

Mass spectrometry-based screening reveals inhibitors of cholesterol 25-hydroxylase

Atikur Rahman[2,3], Elijah H. Hayes[2,3], Drew J. Adams*[1,2,3]

1 Department of Genetics and Genome Sciences, Case Western Reserve University School of Medicine, Cleveland, Ohio 44106, USA.

2 Chemical Biology Program, Case Western Reserve University School of Medicine, Cleveland, Ohio 44106, USA

3 Department of Pharmacology, Case Western Reserve University School of Medicine, Cleveland, Ohio 44106, USA.

Table of Contents

- Figure S1……………………………………………………………………………...S2
- Table S1………………………………………………………………………………S3

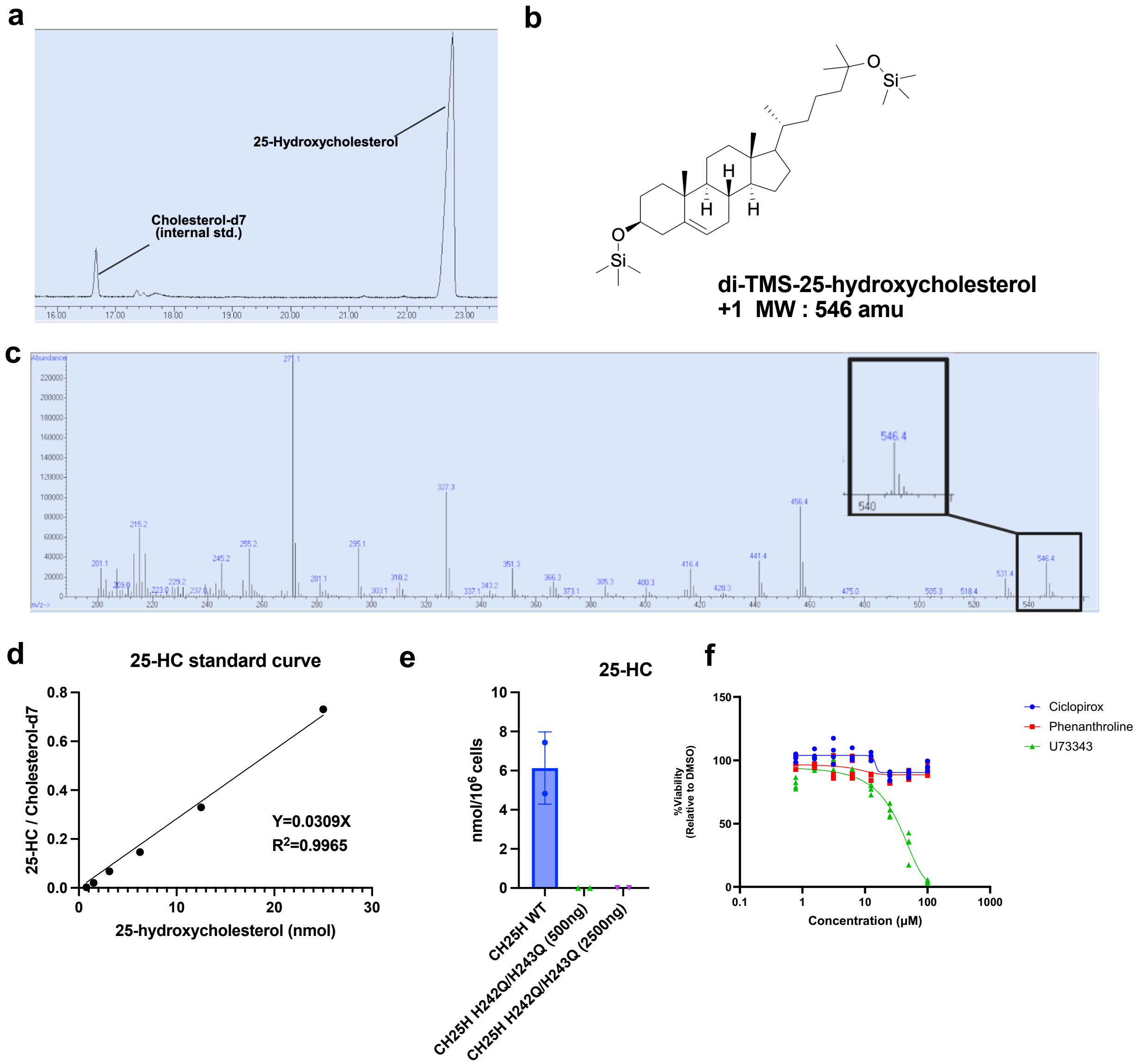

Figure S1. GC-MS method development for the quantification of 25-hydroxycholesterol in cells. **(a)** Representative GC-MS chromatogram delineating peaks of both the internal standard Cholesterol-d7 and 25-hydroxycholesterol. **(b)** Molecular structure and diagnostic ion mass of di-TMS-25-hydroxycholesterol. **(c)** GC-MS ion trace of purified 25-HC highlighting the diagnostic fragment (m/z +1 = 546.4) used for quantitation. **(d)** Standard curve of 25-hydroxycholesterol used to quantify 25-HC levels in cells. **(e)** GC-MS quantitation of 25-HC in HEK293T cells expressing either WT CH25H-FLAG or catalytically dead CH25H H242Q/H243Q-FLAG (500 ng or 2500 ng plasmid).**(f)** Cell viability (CellTiter-Glo, 24 h) in HEK293T cells transiently expressing Ch25h-FLAG following treatment with inhibitors.

Iron chelator library member	Run 1	Run 2	Sterol library member	Run 1	All runs: 25HC/Cholesterol (% of DM		
Licofelone	226.9586	164.9642	Mifepristone	157.3839			
Diclofenac sodium	170.031	206.929	Indomethacin	149.4896			
L2-B	228.0318	108.8987	Ketorolac Tris Salt	138.319			
Triflusal	164.9317	136.7069	Epiandrosterone	128.3898			
Deferasirox	153.823	134.2129	Stigmasterol	128.1879			
Ofloxacin	146.9574	126.988	Rofecoxib	127.328			
Tolfenamic acid	103.0433	149.3896	Betulinic Acid	124.2212			
Zileuton	62.96153	185.1858	Meloxicam Sodium	124.195			
1-Aminobenzotriazole	129.568	117.3621	SC58125	124.1465			
Tenidap	119.4087	127.1369	Acetylsalicylic Acid	122.4905			
1-Phenyl-3-(2-thiazolyl)-2-thiourea	127.747	104.0191	Ganaxolone	122.1324			
Diclofenac Diethylamine	96.81498	123.4895	5α-Pregnan-3α-ol-20-one	120.418			
Guanabenz	96.61821		Zaltoprofen	117.0252			
Actinonin	63.97252	125.3679	Celecoxib	114.5881			
Galeterone	85.15058	91.65859	Fusidic acid sodium	113.6877			
Ketoconazole	96.87825	73.27123	Ibuprofen	111.0552			
Bufexamac	87.62205	80.48705	Dutasteride	107.0106			
Piroxicam	62.76972	76.54169	Lithocholic Acid	106.9825			
BW B70C	62.7993	63.24273	Spironolactone	106.5927			
Mimosine	68.41503	48.81513	Asaraldehyde	105.9168			
Ilomastat	49.38882	66.90806	Sibuprofen	105.4754			
Naproxen	48.9236	63.90702	Cyproterone Acetate	105.3103			
Caffaic Acid	53.65069	55.42378	Asiatic Acid	104.9069			
NNGH	56.07656	51.49309	Phenacetin	103.9495			
Sulindac	55.83124	50.84098	AZ960	103.4431			
Arvanil	66.31214	39.83878	Pregnenolone	103.2341			
Tranilast	36.61094	45.48298	Formestane	103.1246			
GSK-J4	32.39196	44.72175	Lornoxicam	100.7518			
Ketoprofen	33.48483	39.85048	Oxymetholone	98.60557			
Etodolac	33.44392	37.90773	Ruxolitinib	98.47537			
Cyproterone acetate	32.6393	30.95762	SB-590885	98.26767			
Tosedostat	29.58634	32.11897	Rocuronium Bromide	97.986			
Niflumic acid	33.53631	25.63588	S3I-201	97.76253			
Mefenamic acid	23.9728	32.63338	Lumiracoxib	95.48149			
Nimesulide	20.89117	28.98317	Chenodeoxycholic Acid	94.45425			
Ciclopirox	0	0	Oleanolic Acid	94.17937			
1,10-Phenanthroline	0	0	Ursadiol	93.68407			
			Vecuronium Bromide	92.96455			
			Nabumetone	89.41905			
			Trilostane	89.09378			
			Galeterone	88.20436			
			Exemestane	86.66385			
			Danazol	85.96671			
			Pancuronium Bromide	85.89988			
			Valdecoxib	85.64639			
			SC-236	85.51997			
			Ketorolac	83.47078			
			Hyodeoxycholic Acid	80.98997			
			Estrone	80.917			
			Ampiroxicam	80.46028			
			Flunixin Meglumin	79.58559			
			Progesterone	76.87898			
			Carprofen	76.21301			
			Megesterol Acetate	75.6948			
			A861	75.68906			
			3a,21-Dihydroxy-5a-pregnan-20-one	75.50143			
			17α-Hydroxyprogesterone	72.70668			
			Estradiol	70.48682			
			Finasteride	70.09602			
			Acemetacin	69.31896			
			N-p-Tosyl-L-phenylalanine	69.04295			
			LY2784544	68.10847			
			Cryptotanshinone	66.64757			
			NVP-BSK805	61.23073			
			Ethinyl Estradion	57.77747			
			20-Hydroxyecdysone	57.46281			
			Abiraterone Acetate	55.22946			
			Cortexolone	50.78467			
			Meclofenamic Acid Sodium	45.97561			
			Ergosterol	45.18581			
			Estriol	44.24442			
			Pregnenolone Sulfate Sodium	38.1195			
			Dehydroepiandrosterone	37.00823			
			AT9283	34.39594			
			Dioscin	33.7061			
			Enoxolone	29.90828			
			Pregnenolone	29.01616			
			U-73343	4.792954			

Table S1. Raw screening data for the iron chelator and sterol library members.
